# Comparison of an *in house* and a commercial real-time polymerase chain reaction targeting *Toxoplasma gondii* RE gene using various samples collected from patients in Turkey

**DOI:** 10.1186/s12879-019-4666-z

**Published:** 2019-12-10

**Authors:** Mert Döşkaya, Hüsnü Pullukçu, Muhammet Karakavuk, Esra Atalay Şahar, Mehmet Sezai Taşbakan, Meltem Işıkgöz Taşbakan, Mümtaz Yılmaz, Hüseyin Can, Aysu Değirmenci Döşkaya, Adnan Yüksel Gürüz

**Affiliations:** 1 Department of Parasitology, Ege University Faculty of Medicine, 35100 İzmir, Bornova Turkey; 2 Department of Infectious Diseases, Ege University Faculty of Medicine, İzmir, Bornova Turkey; 3 Ege University, Ödemiș Vocational High School, İzmir, Ödemiș Turkey; 4 Department of Biotechnology, Ege University Faculty of Engineering, İzmir, Bornova Turkey; 5 Department of Chest Diseases, Ege University Faculty of Medicine, İzmir, Bornova Turkey; 6 Department of Internal Medicine, Ege University Faculty of Medicine, İzmir, Bornova Turkey; 7 Department of Biology, Molecular Biology Section, Ege University Faculty of Sciences, İzmir, Bornova Turkey

**Keywords:** *Toxoplasma gondii*, RE gene, Diagnosis, Real time PCR, *In house*

## Abstract

**Background:**

*Toxoplasma gondii* is an opportunistic protozoan parasite that can infect all warm-blooded animals including humans and cause serious clinical manifestations. Toxoplasmosis can be diagnosed using histological, serological, and molecular methods. In this study, we aimed to detect *T. gondii* RE gene in various human samples by *in house* and commercial real time polymerase chain reactions.

**Methods:**

A total of 38 suspected cases of toxoplasmosis [peripheral blood (n:12), amnion fluid (n:11), tissue (n:9), cerebrospinal fluid (n:5), and intraocular fluid (n:1)] were included to the study. An *in house* and a commercial RT-PCR were applied to investigate the *T. gondii* RE gene in these samples.

**Results:**

The compatibility rate of the two tests was 94.7% (37/38). When the commercial RT-PCR kit was taken as reference, the sensitivity and specificity of *in house* RT-PCR test was 87.5 and 100%. When the *in house* RT-PCR test was taken as reference, the commercial RT-PCR kit has 100% sensitivity and 96.8% specificity. Incompatibility was detected in only in a buffy coat sample with high protein content.

**Conclusions:**

Both the commercial and *in house* RT-PCR tests can be used to investigate *T. gondii* RE gene in various clinical specimens with their high sensitivity and specificity. In house RT-PCR assay can be favorable due to cost savings compared to using the commercial test.

## Background

*Toxoplasma gondii* is an opportunistic protozoan parasite that can infect all warm-blooded animals including birds and can cause serious clinical manifestations. *T. gondii* causes congenital toxoplasmosis leading to fetal anomalies in newborns, retinochoroiditis causing blindness, deadly toxoplasmic encephalitis in immunocompromised patients or transplant recipients [1, 2].

Toxoplasmosis can be diagnosed using histological, serological, and molecular methods [1, 3–6]. In recent years, serological and molecular diagnostic methods are frequently used. Serological screening for toxoplasmosis is performed in pregnant women in most of the countries during the first trimester. When IgM positivity and low IgG avidity are detected in the pregnant woman, there is a risk of congenital toxoplasmosis in the fetus [1]. In order to rule out this suspicion, amniocentesis is recommended during the 16-18th weeks of gestation and amniotic fluid is investigated with Polymerase chain reaction (PCR) to detect *T. gondii* DNA [1, 7]. In addition, molecular techniques become important due to decreased levels of antibodies in immune suppressed patients [8–10].

The majority of *T. gondii* strains from most parts of the world belong to three different clonal clade, called type I, type II and type III. There are also atypical and recombinant strains present [11–15]. The *T. gondii* B1 gene (GenBank no: AF179871) containing 35 copies, the RE gene (GenBank no: AF146527) containing 200–300 copies and the SAG1 (GenBank: M23658) gene consisting of single copy were used in molecular diagnostic tests [16–19]. In a recent study, the copy numbers for the B1 gene were found to be 5, 12 and 7 times lower than the previous estimations of 35 copies for type I, type II and Type III strains, respectively. For *T. gondii* RE gene were found to be 8, 4 and 4 times lower than the previous estimations of 200–300 copies for type I, type II and Type III strains, respectively [17]. PCR studies performed in various clinical samples showed that *T. gondii* RE gene was more sensitive and specific than B1 gene [20–29]. Moreover *Toxoplasma* Reference centers in Europe are using PCR reactions targeting *T. gondii* RE gene more frequently compared to *T. gondii* PCR targeting B1 gene [30, 31].

In this study, two hybridization probe based methods which are an *in house* Real Time PCR and a commercial Real Time PCR targeting *T. gondii* RE gene were compared in terms of sensitivity and specificity using different clinical samples.

## Methods

### Clinical samples

In this study, 38 specimens [12 peripheral blood sample, 11 amniotic fluid, 9 tissue, 5 cerebrospinal fluid (CSF) and 1 intraocular fluid] obtained from 38 clinically toxoplasmosis suspected seropositive patients admitted to Ege University Faculty of Medicine, were investigated for the presence of *T. gondii* using an *in house* Real Time PCR and a commercial Real Time PCR targeting RE gene.

### Specimen processing and DNA extraction

DNA extraction from amniotic fluid, peripheral blood, tissue, CSF, or intraocular fluid sample was performed using the Qiagen Mini Kit according to the manufacturer’s protocol. Initially, specimens were processed. During the DNA extraction of blood sample, the buffy coat part was used [31–33]. For this purpose, 2–4 ml whole blood in EDTA was centrifuged at 3000 rpm for 15 min. After centrifugation, plasma was discharged until ~ 300 μl plasma remained. Then, 500 μl of sample was used for DNA extraction [~ 300 μl plasma (+) ~ 100 μl buffy coat (+) ~ 100 μl of erythrocyte cluster]. During the extraction of DNA from amniotic fluid, at least 6 ml sample was used [34]. For a sample more than 6 ml, whole specimen was transferred to a sterile 15 ml tube and centrifuged at 3000 rpm for 15 min. Next, the supernatant was discarded until ~ 6 ml amniotic fluid remained. For the tissue, 50 mg sample was finely chopped and added to tube containing ATL buffer, 80 μl Proteinase K, 0,1 mm glass beads, and 2 mm zirconia beads (BioSpec Products, U.S.A.) and incubated for 15 min at 56 °C with 1400 rpm in a heat shaker (Lab4You) until the sample melted. Next, the tissue samples were incubated at 95 °C for 5 min. After incubation, samples were vortexed for 2 min on a Disruptor Genie (Scientific Industries, U.S.A.) [35, 36]. CSF and intraocular fluid samples were used directly when the volume of sample was ~ 200 μl, and in the case of excess, the sample was centrifuging at 3000 rpm and the supernatant was discarded until ~ 200 μl sample remained.

### Real time PCR

*In house* Real Time PCR to detect 134 bp region of *T. gondii* RE gene (GenBank no: AF146527) was performed as described with some minor modifications [20]. The analysis of results was performed by 1.5 LightCycler Real Time instrument using LightCycler software, Version 3.5 according to the manufacturers protocol (Roche). Twenty microliter final volume PCR reaction included 1x LightCycler Fast Start DNA Master HybProbe mix with MgCl_2_ final concentration adjusted to 5 mM (Roche), the primers 5′-AGGCGAGGGTGAGGATGA-3′ (18 nt, TOX-SE forward primer; final concentration: 0.5 μM) and 5′-TCGTCTCGTCTGGATCGCAT-3′ (20 nt, TOX-AS reverse primer; final concentration: 0.5 μM), the hybridization probes 5′-GCCGGAAACATCTTCTCCCTCTCC-3′-FL (24 nt, TOX FLU, labeled at the 3′ end with fluorescein; final concentration: 0.1 μM) and 5′-640-CTCTCGTCGCTTCCCAACCACG-3′ (22 nt, TOX LCR labeled at the 5′ end with LC-Red 640; final concentration: 0.5 μM) (IDT), DNA template or controls. The PCR amplification reactions were performed by the following calculated protocol: 10 min initial denaturation step at 95 °C, followed by 50 cycles of 5 s at 95 °C, 10 s at 60 °C, and 15 s at 72 °C. PCR reactions were performed in duplicate.

As positive and negative controls, *T. gondii* genomic DNA serially 10-fold diluted ranging from 10^6^ to 10^1^ parasites/μl and distilled water were used, respectively. In clinical samples and positive controls, crossing point (Cp) values were used to assess the amount of *T. gondii* DNA. Melting curve analysis was performed using the following calculated protocol: 20 s denaturation step at 95 °C followed by 20 s annealing step at 40 °C and extension step gradually increasing temperature to 85 °C.

To analyze the PCR inhibition attributable to the specimen matrix, PCR reactions were prepared in duplicate for each sample. One of the reactions contained only purified patient DNA sample and the other reaction contained 1 *T. gondii* tachyzoite spiked into the purified patient sample. The sample is accepted as inhibited when the spiked control did not show any crossing point (Cp) value.

The commercial *T. gondii* Real Time PCR kit (TIBMolBiol) targeting 134 bp fragment of *T. gondii* RE gene was applied according to the manufacturer’s protocol. The analysis of results was performed by 1.5 LightCycler Real Time instrument using LightCycler software, Version 3.5 according to the manufacturers protocol (Roche). In this test, the internal control target was investigated as multiplex. The control reaction mix contained the primer and the probe mix that determines the internal control, while the reaction also included the lambda phage as the extraction control target. Twenty microliter final volume PCR reaction included 1x LightCycler Fast Start DNA Master HybProbe mix with MgCl_2_ final concentration adjusted to 4.5 mM (Roche), 2 μl PSR mix (parameter specific reagents; containing *T. gondii* RE gene specific primers and hybridization probes), DNA template or controls, 2 μl control target (lambda phage) and 0.5 μl control reaction mix (contains primers and probe for lambda phage). The PCR amplification reactions were performed by the following calculated protocol: 10 min initial denaturation step at 95 °C, followed by 50 cycles of 5 s at 95 °C, 5 s at 62 °C, and 15 s at 72 °C. Moreover, a single cycle of 55 °C with 0.5 step size was included to the amplification step. PCR reactions were performed in duplicate.

As positive and negative controls, *T. gondii* plasmid containing 134 bp fragment of RE gene serially 10-fold diluted ranging from 10^6^ to 10^1^ copy plasmids/reaction and distilled water were used. In clinical samples and positive controls, crossing point (Cp) values were used to assess the amount of *T. gondii* DNA. Melting curve analysis was performed using the following calculated protocol: 20 s denaturation step at 95 °C followed by 20 s annealing step at 40 °C and extension step gradually increasing temperature to 85 °C.

## Results

According to the results, among the 38 clinical samples analyzed, *T. gondii* RE gene was not detected in 30 of them and *T. gondii* RE gene was detected in 6 samples by both *in house* Real Time PCR and commercial Real Time PCR (Table 1). Regarding the remaining two samples; one sample was partly compatible in both assays in which *T. gondii* RE gene was detected during the first run and not detected in the subsequent using *in house* Real Time PCR, whereas the commercial Real Time PCR was positive in both runs for this sample. In the remaining sample, the results were completely incompatible between both assays (Table 1). Both of these samples were buffy coat samples. As a result, the compatibility rate of both tests was 97.4% (37/38) after two tests.

**Table 1:** Comparison of in house and commercial Real Time PCR kits targeting *T. gondii* RE gene in 38 clinical samples

Sample type	*in house* Real Time PCR	Commercial Real Time PCR	Compatibility	Number of sample with the same PCR results
Peripheral blood (Buffy coat)	−/− +/+ −/− +/−	−/− +/+ +/+ +/+	Compatible Compatible Partly compatible Completely incompatible	8 2 1 1
Amniotic fluid	−/− +/+	−/− +/+	Compatible Compatible	10 1
Tissue	−/− +/+	−/− +/+	Compatible Compatible	6 3
Cerebrospinal fluid (CSF)	−/−	−/−	Compatible	5
Intraocular fluid	−/−	−/−	Compatible	1
			TOTAL	38

The sensitivity of the *in house* Real Time PCR was 87.5% (7/8) and the specificity was 100% (30/30) when we take commercial Real Time PCR as the golden standard. Conversely, if we take *in house* Real Time PCR as the golden standard, the sensitivity and specificity of the commercial Real Time PCR was 100% (7/7) and 96.8% (30/31), respectively. Due to low sample size, individual sensitivity and specificity values were not calculated.

As the results of the melting curve analysis of positive control and positive clinical samples were examined, it was found that Tm of all samples peaked around 67.5 ± 1 °C using *in house* and commercial Real Time PCRs. When the internal control data were examined, any inhibition was not detected in both tests. As the positive controls were examined, it was found that the analytical sensitivity of the commercial Real Time PCR was 10 copy plasmid/reaction and the analytical sensitivity of the *in house* Real Time PCR was 1 parasite/reaction. No signal was detected in the distilled water negative control samples.

## Discussion

Molecular diagnostic tests have high sensitivity and specificity in diagnosing congenital toxoplasmosis, toxoplasmic retinochoroiditis, as well as organ transplantation and AIDS patients [37]. *T. gondii* B1 and RE genes are the most frequently targeted markers in the molecular diagnosis of toxoplasmosis due to their high copy numbers [16, 17]. Some studies have shown that the *T. gondii* RE gene is more sensitive and specific than the B1 gene [20–30]. In East Africa, blood samples of 63 patients were investigated with Real Time PCR methods targeting the B1 and RE genes of *T. gondii*. Interestingly, B1 gene was detected in all cases known to be positive for *T. gondii*, whereas in 3 samples (4.8%), RE gene could not be detected. The authors stated that the G/C mutation at position 275 of the RE gene inhibited the binding of the primer to the RE gene and affected real time PCR results. In addition, the genotypes detected in Africa are different from Europe. The higher ratio of RE/B1 copy number detected in type II strains which is more common in Europe has been shown to be a cause of the high specificity and success of real time PCR targeting the RE gene in Europe [29].

In Turkey, two cases of congenital toxoplasmosis detected in 1972 and 2007 have been isolated from two infants and has been shown to be similar to the isolates of African genotype 1 [38]. In addition, two studies conducted in big groups of cats and wild birds of Turkey, Type II and III strains are found to be more frequent like in Europe [14, 15]. In our country, more frequent detection of type II strains in animals suggests that PCR targeting the *T. gondii* RE gene may be more sensitive. Thus, in this study, two very similar hybridization probe based methods which are an *in house* Real Time PCR described by Cassaign et al., 2006 and a commercial Real Time PCR kit targeting the same region of *T. gondii* RE gene were first time compared in terms of sensitivity and specificity using various clinical samples.

A total of 38 suspected cases of toxoplasmosis were included to the study. The reason for low sample size can be attributed to presence of *T. gondii* in blood approximately for only 2 weeks during acute infection of immunocompetent patients [1, 2]. Besides, in a patient infected with *T. gondii*, seroconversion (i.e. IgG positivity) takes place around 15 days and as the clinicians order to perform *T. gondii* PCR, tachyzoites may become latent in tissues (also called chronic toxoplasmosis) and not found in blood circulation [1, 2]. In addition, an important clinical presentation called congenital toxoplasmosis is a rare disease according to the Oprhanet report series [39]. Overall, *T. gondii* DNA positivity may not be frequently detected in blood or amniotic fluid samples of toxoplasmosis cases. Thus, although we aimed to evaluate more *T. gondii* DNA positive samples in this study, we couldn’t achieve it and the low *T. gondii* DNA positive sample size can affect the interpretation of the sensitivity ratio which is the limitation of this study.

The results showed that the compatibility rate of the *in house* Real Time PCR and commercial Real Time PCR targeting the *T. gondii* RE gene was 97.4% using 38 different clinical samples. Among the 38 samples analyzed in this study, 36 of them (six positive and 30 negative) were found to be fully compatible, while one buffy coat sample was partly compatible and the other was completely incompatible (Table 1). In these two samples, the UV260/280 ratio was> 1.8 and no inhibition was observed. In one sample, the *in house* Real Time PCR was one time negative and the other time positive which can be attributed to a low *T. gondii* DNA in the sample (Table 1). In this sample, the Cp value of the *in house* Real Time PCR was 36, supporting the difference among the results. Similarly, with the commercial Real Time PCR, the Cp value of 34.6 supports the fact that the concentration of *T. gondii* RE gene was low. Consequently, the commercial Real Time PCR kit can be considered to be more sensitive in detecting the *T. gondii* RE gene. In this situation, the problem of hypersensitivity of the PCR may occur leading to false positivity due to forced cross-linking of primers and probes to primer dimers at the last cycles of the PCR. In this study, the Cp value of the buffy coat sample (which was found two times negative with *in house* Real Time PCR and two times positive with commercial Real Time PCR kit) as detected by commercial Real Time PCR kit were 34.3 and 34 and can be evaluated as hypersensitivity. Conversely, the inaccurate negative result as detected by *in house* Real Time PCR may be due to the loss of sensitivity because of high protein content in buffy coat. In a study, the buffy coat samples of 44 patients with clinically proven acute toxoplasmosis was investigated in two different centers by hybridization probe based Real Time PCR assay targeting the *T. gondii* RE gene. Both centers found 25% (9/44) positivity. Only four of these nine patients were found to be positive in both centers, while four samples were positive in one center and one sample positive in another center [33]. As a result, buffy coat samples can be considered as challenging samples for the detection of *T. gondii* DNA.

## Conclusions

Both tests were compatible with amniotic fluid, tissue, CSF or intraocular fluid samples, and showed complete incompatibility only in a buffy coat sample with a dense protein content. In particular, it may be useful to analyze high protein content samples such as buffy coat twice to detect low amount of *T. gondii* DNA. As a result, the high compatibility, analytical sensitivity, sensitivity, and specificity of both tests suggest that the Real Time PCR methods targeting *T. gondii* RE gene can be used to investigate *T. gondii* DNA in various clinical samples. *In house* real time PCR can be preferred by developing laboratories due to cost savings.

## Data Availability

The dataset analyzed during the current study is available from the corresponding author on reasonable request.

## Abbreviations

CSF: Cerebrospinal fluid
DNA: Deoxyribonucleic acid
IgG: Immune globulin G
IgM: Immune globulin M
min: minutes; rpm: rounds per minute
PCR: Polymerase chain reaction
RE: Repeat element
RT-PCR: Real-time polymerase chain reaction
*Toxoplasma gondii*: *T. gondii*
